# Assessment of Three New Loci from Genome-wide Association Study in Essential Tremor in Chinese population

**DOI:** 10.1038/s41598-017-08863-5

**Published:** 2017-08-11

**Authors:** Yuan Zhang, Yuwen Zhao, Xiaoting Zhou, Kai Li, Minhan Yi, Jifeng Guo, Xinxiang Yan, Beisha Tang, Qiying Sun

**Affiliations:** 1 Department of Neurology, Xiangya Hospital, Central South University, Changsha, Hunan 410008 China; 2 Department of Geriatrics, Xiangya Hospital, Central South University, Changsha, Hunan 410008 China; 3 State Key Laboratory of Medical Genetics, Changsha, Hunan 410078 China; 4 Institute of Information Security and Big Data, Central South University, Changsha, Hunan 410083 China; 5 National Clinical Research Center for Geriatric Diseases, Changsha, Hunan 410078 China; 6 Key Laboratory of Hunan Province in Neurodegenerative Disorders, Central South University, Changsha, Hunan 410008 China

## Abstract

Essential tremor (ET) is the most common tremor disorder. Evidences indicated that genetics plays an essential role in the researches of etiology. A new genome-wide association study (GWAS) from European population identified three novel loci in ET, which were rs10937625 in *STK32B*, rs17590046 in *PPARGC1A*, and rs12764057, rs10822974 and rs7903491 in *CTNNA3*. Due to the different genetic background in different population, we performed a case-control study to investigate these variants in a cohort of 533 subjects in Chinese population. We found a significant difference in the distributions of genotypes and alleles frequencies between ET and control groups of rs10937625 (genotype *p* = 0.037, OR = 0.69[0.48–0.98]; allele *p* = 0.033, OR = 0.82[0.69–0.99]) and rs7903491 (genotype *p* = 0.030, OR = 1.34[1.03–1.74]; allele *p* = 0.029, OR = 1.16[1.02–1.32]) after adjusted for age and gender. And no associations were detected between rs17590046 (genotype *p* = 0.794; allele *p* = 0.791), rs12764057 (genotype *p* = 0.337; allele *p* = 0.337), rs10822974 (genotype *p* = 0.102; allele *p* = 0.100) and ET in Chinese population individually. Our research supports that C allele of rs10937625 in *STK32B* is a protective factor and G allele of rs7903491 in *CTNNA3* is a risk factor for ET in Chinese population.

## Introduction

Essential tremor (ET) is the most common tremor disorder with the prevalence of 0.9% at all ages, 4.6% at age ≥ 60~65 years and 21.7% at age ≥ 95 years^1^ respectively. It is characterized by postural and kinetic tremor of hands, arms, head, voice and leg, especially upper extremities^2^. Except for tremor, there are many other non-motor impairments reported in ET patients^3^ such as cognitive abnormalities, dementia, anxiety and depression, sleep disorder, and subjective hearing impairment. Despite its high prevalence and various symptoms, it is not clearly understood on the etiology of ET. Therefore, the research of ET should not be underestimated.

Although the etiology is unclear, the high prevalence of positive family history of ET supports that genetics plays an essential role in ET and several loci and genes are linked to ET^4^. In 2009, a genome-wide association study (GWAS) identified that SNP rs9652490 in *LINGO1* ^5^ was associated with ET in Icelandic, European and American populations. In 2012, another GWAS study from European population found that SNP rs3794087 of *SLC1A2* ^6^ was related to ET. Through the method of exome sequencing, a pathogenic variant of *FUS* ^7^ was identified in a large ET family of French-Canadian origin in 2012. Similarly, pathogenic variants in *HRTA2* ^8^ and *TENM4* ^9^ were individually found in ET in a six-generation consanguineous Turkish kindred in 2014 and families of Spanish origin in 2015. However, these reported results were not always consistent in Chinese population^10–14^ due to different genetic background and research method.

Recent, a new GWAS of 2807 ET patients and 6447 controls from European population has identified three new susceptibility loci associated with ET^15^. They are rs10937625 in serine/threonine kinase *STK32B*, rs17590046 in the transcriptional coactivator *PPARGC1A* and rs12764057, rs10822974 and rs7903491 in the cell-adhesion molecule *CTNNA3*. Notably, the variant rs17590046 in *PPARGC1A* was replicated in an cohort of Asian population from Singapore^16^, which indicated that rs17590046 may be a common genetic factor for ET patients from Asian and European populations. Due to the different genetic background of different populations, we conducted a case-control study to investigate the association between the three new loci and ET in a cohort of Chinese population.

## Results

The characteristics of all participants included in this study were described in Table 1. All five variants selected were in agreement with HWE in groups of both cases and controls (*p* > 0.05). Genotypes and alleles distributions of the five polymorphisms in cases and controls were shown in Table 2. Statistically significant differences of genotypes and alleles frequencies for rs10937625 in *STK32B* (genotype *p* = 0.037, OR = 0.69[0.48–0.98]; allele *p* = 0.033, OR = 0.82[0.69–0.99]) were detected between ET patients and healthy controls, which indicate a protective role of rs10937625 in *STK32B* to ET. Besides, there were significant differences of rs7903491 in *CTNNA3* (genotype *p* = 0.030, OR = 1.34[1.03–1.74]; allele *p* = 0.029, OR = 1.16[1.02–1.32]) between ET cases and controls, which support a risky role of rs7903491 in *CTNNA* to ET. However, no associations were observed between the other three variants and ET.

**Table 1:** Demographic and clinical characteristics of all subjects.

Variables	ET patients	HCs
Total Numbers	218	315
Gender, Male/Female (%)	113/105(51.8/48.2)	168/147(53.3/46.7)
Family history, Positive/Negative	157/61	—
Age at examination (years)	46.73 ± 17.04	36.87 ± 10.46
Age at onset (years)	36.23 ± 15.93	—
EOET/LOET	118/100(54.1/45.9)	—

Age in the table were presented in mean and standard deviation (SD). We chose the onset age of 40 years as the cut-point to define EOET and LOET. Abbreviations: ET: Essential tremor; HCs: health controls; EOET: early onset Essential tremor; LOET: late onset Essential tremor.

**Table 2:** Distributions of genotypes and alleles frequencies of five variants in ET patients and healthy controls.

Variants	Genotypes/alleles	ET patients (n = 218) (%)	HCs (n = 315) (%)	*p* OR(95%CI)
rs10937625	CC	3(1.4)	15(4.8)	**0**.**037 0**.**69[0**.**48–0**.**98]**
	TC	52(23.9)	91(28.9)	
	TT	163(74.8)	209(66.3)	
	C	58(13.3)	121(19.2)	**0**.**033 0**.**82[0**.**69–0**.**99]**
	T	378(86.7)	509(80.8)	
rs17590046	CC	2(0.9)	4(1.3)	0.794 1.06[0.68–1.67]
	TC	33(15.1)	43(13.7)	
	TT	183(83.9)	268(85.1)	
	C	37(8.5)	51(8.1)	0.791 1.03[0.82–1.30]
	T	399 (91.5)	579(91.9)	
rs12764057	GG	14(6.4)	18(5.7)	0.337 1.16[0.86–1.55]
	TG	88(40.4)	115(36.5)	
	TT	116(53.2)	182(57.8)	
	G	116(26.6)	151(24.0)	0.337 1.08[0.93–1.25]
	T	320(73.4)	479(76.0)	
rs10822974	AA	36(16.5)	79(25.1)	0.102 0.81[0.62–1.04]
	GA	116(53.2)	148(47.0)	
	GG	66(30.3)	88(27.9)	
	A	188(43.1)	306(48.6)	0.100 0.90[0.79–1.02]
	G	248(56.9)	324(51.4)	
rs7903491	GG	44(20.2)	46(14.6)	**0**.**030 1**.**34[1**.**03–1**.**74]**
	AG	105(48.2)	148(47.0)	
	AA	69(31.7)	121(38.4)	
	G	193(44.3)	240(38.1)	**0**.**029 1**.**16[1**.**02–1**.**32]**
	A	243(55.7)	390(61.9)	

All ET patients were compared with all healthy controls, and *p* values, estimated ORs and 95%CI were determined using binary logistic regression adjusted for age and gender. Significant p values <0.05 are shown in bold. Abbreviations: ET: Essential tremor; HC: healthy control; OR: odds ratio; 95%CI: 95% confidence interval.

After age stratification, in late onset ET (age at onset ≥ 40 years) patients and controls, the differences of genotypes attributions and alleles frequencies were still significant in rs10937625 of *STK32B* (genotype *p* = 0.002, OR = 0.41[0.24–0.71]; allele *p* = 0.001, OR = 0.64[0.49–0.83]). However, it is not the situation in ET of the other four variants (Table 3). When stratified by gender, there were no large differences in the allele frequencies of the five variants between both groups (Supplemental Table S1). What’s more, there were no significant differences in the genotypes and allele attributions of the five polymorphisms in ET patients with family history and controls (Supplemental Table S2).

**Table 3:** Genotypes and alleles distributions of five selected variants in ET and controls stratified by age at onset.

Variants	Genotypes/Alleles	EOET/LOET (118/100) (%)	HC<40/HC ≥ 40 (203/112) (%)	*p* OR(95%CI)^a^	*p* OR(95%CI)^b^
rs10937625	CC	2(1.7)/1(1.0)	6(3.0)/9(8.0)	0.997 1.00[0.61–1.65]	**0**.**002 0**.**41[0**.**24–0**.**71]**
	TC	28(23.7)/24(24.0)	53(26.1)/38(33.9)		
	TT	88(74.6)/75(75.0)	144(70.9)/65(58.0)		
	C	32(13.6)/26(13.0)	65(16.0)/56(25.0)	0.997 1.00[0.78–1.29]	**0**.**001 0**.**64[0**.**49–0**.**83]**
	T	204(86.4)/174(87.0)	341(84.0)/168(75.0)		
rs17590046	CC	0(0)/2(2.0)	4(2.0)/0(0)	0.360 0.75[0.40–1.40]	0.090 1.91[0.91–4.03]
	TC	18(15.3)/15(15.0)	30(14.8)/13(11.6)		
	TT	100(84.7)/83(83.0)	169(83.3)/99(88.4)		
	C	18(7.6)/19(9.5)	38(9.4)/13(5.8)	0.344 0.86[0.62–1.18]	0.084 1.39[0.96–2.02]
	T	218(92.4)/181(90.5)	368(90.6)/211(94.2)		
rs12764057	GG	7(5.9)/7(7.0)	13(6.4)/5(4.5)	0.769 0.94[0.63–1.41]	0.059 1.57[0.98–2.53]
	TG	47(39.8)/41(41.0)	80(39.4)/35(31.3)		
	TT	64(54.2)/52(52.0)	110(54.2)/72(64.3)		
	G	61(25.8)/55(27.5))	106(26.1)/45(20.1)	0.770 0.97[0.79–1.19]	0.058 1.26[0.99–1.59]
	T	175(74.2)/145(72.5)	300(73.9)/179(79.9)		
rs10822974	AA	19(16.1)/17(17.0)	53(26.1)/26(23.2)	0.497 0.88[0.62–1.26]	0.123 0.73[0.49–1.09]
	GA	67(56.8)/49(49.0)	90(44.3)/58(51.8)		
	GG	32(27.1)/34(34.0)	60(29.6)/28(25.0)		
	A	105(44.5)/83(41.5)	196(48.3)/110(49.1)	0.495 0.94[0.79–1.12]	0.124 0.86[0.70–1.04]
	G	131(55.5)/117(58.5)	210(51.7)/114(50.9)		
rs7903491	GG	20(16.9)/24(24.0)	32(15.8)/14(12.5)	0.406 1.17[0.81–1.68]	0.089 1.41[0.95–2.09]
	AG	63(53.4)/42(42.0)	97(47.8)/51(45.5)		
	AA	35(29.7)/34(34.0)	74(36.5)/47(42.0)		
	G	103(43.6)/90(45.0)	161(39.7)/79(35.3)	0.412 1.08[0.90–1.29]	0.077 1.20[0.98–1.47]
	A	133(56.4)/110(55.0)	245(60.3)/145(64.7)		

*P* values were determined using binary logistic regression adjusted for age and gender, and p values <0.05 indicate significant differences between two groups. Abbreviations: ^a^means EOET compared with healthy controls <40 years. ^b^means LOET compared with healthy controls ≥40 years. ET: essential tremor; HC: healthy controls; EOET: early onset essential tremor related to age at onset <40 years; LOET: late onset essential tremor related to age at onset ≥40 years.

## Discussion

We conducted a case control study to explore the GWAS-linked three new loci and ET from Chinese population. Fortunately, we found significant differences of genotypes and alleles distributions between ET subjects and controls of rs10937625 in *STK32B* and rs7903491 in *CTNNA3* in Chinese population, which may help us understand the genetic background of ET in Chinese.

SNP rs10937625 locates in an intronic region of *STK32B*, which codes for serine/threonine kinase and its biological function is unknown. From the bioinformatics, rs10937625 is located in a DNase hypersensitive place of *STK32B*, which may participant in regulation of STK32B expression. Based on the clinicopathological assessment^15^ of brain tissues, it was found that the expression of STK32B was significantly higher in ET patients’ brains than controls’. Our results showed that allele C, the minor allele of rs10937625 in *STK32B*, had a significantly lower frequency in ET patients (13.3%) in comparison with controls (19.2%). The protective minor allele C of rs10937625 is associated with reduced expression of STK32B in cerebellar cortex in the Braineac QTL database^17^. All these evidences further support a protective role of C allele of rs10937625 in *STK32B* to ET. And the association of rs10937625 in ET from Chinese population was consistent with that in European population. However, Bin Xiao *et al*.^16^ reported that there were no associations between rs10937625 and ET in a cohort from Singapore. Instead, significant difference with ET was observed in rs17590046 in Xiao’s study. Rs17590046 is in an intronic region of *PPARGC1A* encoding the peroxisome proliferator-activated receptor gamma coactivator 1-alpha (PGC-1α). PGC-1α can regulate mitochondrial number and function in response to external stimuli, which take part in the pathogenic process of neurodegenerative diseases^18, 19^. In contrast to the reported protective role of the minor allele C in European population^15^ and Asian from Singapore^16^, we found no association between rs17590046 and ET in our cohort. In addition, we found rs7903491 in *CTNNA3* as an ET risk factor in our Chinese cohort. *CTNNA3* codes for catenin alpha 3 which is a cell-cell adhesion molecule. Based on a genetic analysis study of 1313 late onset Alzheimer’s disease (LOAD) and 1449 healthy controls, Miyashita, A., *et al*.^20^ reported that *CTNNA3* may influence the development of LOAD through a female-specific mechanism. A large population-based design^21^ was conducted to explore the risk of developing AD or ET among families of PD patients and healthy controls. This study reported that the risk of AD was over three times in probands of younger onset PD than healthy controls’ relatives. And the risk of ET was significantly increased in families of PD cases than that in relatives of healthy controls. All these evidences indicate that these three common neurodegenerative diseases of ET, AD and PD may be related in some way, sharing genetic predisposition and environmental exposure, even common pathophysiological mechanisms.

Till now, there are three GWAS studies in ET. In 2009, the first GWAS study in ET identified that rs9652490 in *LINGO1* ^5^ was related with ET in Icelandic, European and American populations. In 2012, the second GWAS research in ET from European population found that rs3794087 of *SLC1A2* ^6^ was associated to ET. Comparing to the previous two GWAS studies (452/436 ET patients per discovery stage), this new GWAS study in European population^15^ used the largest number of samples (1778 ET patients and 5376 controls) in the discovery stage and were further replicated in an independent cohort of 1029 ET patients and 1065 controls. And unfortunately, the third GWAS study failed to replicate the results of the former two GWAS studies. The reasons maybe correlated with the number of participants and research method.

The reasons related to the different results from our study and other two publications can be divided into three parts. Firstly, there are ethnic differences between Caucasian and Asian populations. This difference even exists between Chinese and non-Chinese in Asian. Secondly, the clinical heterogeneity of ET samples included may contribute to the genetic difference. For instance, there are a larger percentage of family ET in our study compared with Bin’s study, and the mean age is younger than the other two studies. Thirdly, the sample size in this study is smaller in comparison with the other two studies. With the similar positive odds ratio and minor allele frequency of all variants to that in other databases and publications (Supplemental Table S3), it was believed that the results in our study can be an evidence for further study in other populations.

A limitation of this study is that the sample size could be larger. Another limitation is that we did not analyze the association of the five polymorphisms and sporadic ET. Thus, we could not distinguish the genetic contribution of the five polymorphisms to family ET and sporadic ET. This study had several strengths. First, all ET patients and controls were all clearly diagnosed. Second, the genotyping and statistic methods were reliable.

In conclusion, we found C allele of rs10937625 in *STK32B* is a protective factor and G allele of rs7903491 in *CTNNA3* is a risk factor for ET in Chinese population. And no associations were detected between rs17590046 in *PPARGC1A*, rs12764057 and rs10822974 in *CTNNA3* with ET in Chinese population. Further researches in other populations as well as functional studies are needed in order to explore the precise role of these variants in the pathogenesis of ET.

## Methods

### Participants

This research was conducted in agreement with the Declaration of Helsinki. All participants signed up consent before participating in this study approved by the Ethics Committee of Xiangya Hospital, Central South University. Informed consent to publish identifying information was also obtained from all the subjects. The methods in this study were performed in accordance with the approved guidelines.

218 ET patients were recruited from the outpatient neurology of Xiangya Hospital. ET subjects were diagnosed by two or more experienced neurologists according to the Movement Disorder Society (MDS) Consensus Criteria^2^. Patients with psychogenic tremor, hyperthyroidism tremor and other secondary causes of tremor have been excluded in our study samples. We chose the onset age of 40 years as the cut-point to define early onset essential tremor (EOET) and late onset essential tremor (LOET)^22^. A total of 315 healthy controls were included from the Health Examination Centre of Xiangya Hospital. All the recruited participants are Chinese Han individuals by self-report.

### DNA extraction and Genotyping

DNAs of all participants were extracted from peripheral blood according to standard protocol. Genomic DNA was amplified by polymerase chain reaction (PCR) as previously described using forward primers and reverse primers designed. Genotyping of the variants were performed using Sanger sequencing with an ABI3730XL genetic analyzer (Applied Biosystems Inc, Foster City, California, USA).

### Statistical analysis

Hardy-Weinberg equilibrium (HWE) for genotype distribution in patients and controls was examined. Binary logistic regression was performed to compare genotypes and alleles frequencies between case and control groups adjusted for covariates such as age and gender. All tests were 2-tailed, with the significance level set at p < 0.05. All statistics were computed using statistical software SPSS for Windows, version 18.0 (SPSS Inc, Chicago, 2IL, USA).

## Electronic supplementary material

Supplementary Information
