# Supplementary material for: Assessment of Three New Loci from Genome-wide Association Study in Essential Tremor in Chinese population
Source: Sci Rep. 2017 Aug 11;7:7981. doi: 10.1038/s41598-017-08863-5 (PMC5554251; doi:10.1038/s41598-017-08863-5)
Supplement: Supplementary file 1 — Supplementary Information [file 41598_2017_8863_MOESM1_ESM.doc]

# Assessment of Three New Loci from Genome-wide Association Study in Essential Tremor in Chinese population

Yuan Zhang^1^, Yuwen Zhao^1^, Xiaoting Zhou^1^, Kai Li^1^, Minhan Yi^3,4^, Jifeng Guo^1,3,5,6^, Xinxiang Yan^1,5,6^, Beisha Tang^1,3,5,6^, Qiying Sun^2,5,6*^

^1^ Department of Neurology, Xiangya Hospital, Central South University, Changsha, Hunan 410008, China

^2^ Department of Geriatrics, Xiangya Hospital, Central South University, Changsha, Hunan 410008, China

^3^State Key Laboratory of Medical Genetics, Changsha, Hunan 410078, China

^4^ Institute of Information Security and Big Data, Central South University, Changsha, Hunan 410083, China

^5^ National Clinical Research Center for Geriatric Diseases, Changsha, Hunan 410078, China

^6^ Key Laboratory of Hunan Province in Neurodegenerative Disorders, Central South University, Changsha, Hunan 410008, China

^*^ Correspondence author and requests for materials should be addressed to Qiying Sun ([sunqiying550699@sina.com](mailto:sunqiying550699@sina.com)).

**Supplemental Table S1.** **Genotypes and alleles distributions of five selected variants in ET and controls stratified by gender.** *P* values were determined using binary logistic regression adjusted for age, and p values ＜0.05 indicate significant differences between two groups. Abbreviations: a All male ET patients were compared with total male healthy controls. b All female ET patients were compared with all female healthy controls. ET: essential tremor; HC: healthy controls.

| Variants | Genotypes/ Alleles | Males/Females in ET （113 / 105） (%) | Males/Females in HC（168 / 147） (%) | *p*  OR(95%CI) ^a^ | *p* OR[95%CI] ^b^ |
| --- | --- | --- | --- | --- | --- |
| rs10937625 | CC | 2(1.8) / 1(1.0) | 9(5.4) / 6(4.1) | 0.076 0.63[0.38-1.05] | 0.243 0.74[0.45-1.23] |
|  | TC | 23(20.4)/ 29(27.6) | 48(28.6)/ 43(29.3) |  |  |
|  | TT | 88(77.9)/ 75(71.4) | 111(66.1)/ 98(66.7) |  |  |
|  | C | 27(11.9)/31(14.8) | 66(19.6)/55(18.7) | 0.063 0.78[0.60-1.01] | 0.245 0.86[0.67-1.11] |
|  | T | 199(88.1)/ 179(85.2) | 270(80.4)/239(81.3) |  |  |
| rs17590046 | CC | 1(0.9)/ 1(1.0) | 3(1.8)/1(0.7) | 0.750 1.11[0.58-2.13] | 0.731 1.12[0.58-2.16] |
|  | TC | 14(12.4)/ 19(18.1) | 17(10.1)/ 26(17.7) |  |  |
|  | TT | 98(86.7)/ 85(81.0) | 148(88.1)/ 120(81.6) |  |  |
|  | C | 16(7.1)/21(10.0) | 23(6.8)/28 (9.5) | 0.731 1.06[0.75-1.51] | 0.740 1.01[0.77-1.45] |
|  | T | 210(92.9)/ 189 (90.0) | 313(93.2)/266(90.5) |  |  |
| rs12764057 | GG | 4(3.5)/ 10(9.5) | 9(5.4)/ 9(6.1) | 0.466 1.18[0.76-1.81] | 0.592 1.12[0.74-1.69] |
|  | TG | 52(46.0)/ 36(34.3) | 59(35.1)/ 56(38.1) |  |  |
|  | TT | 57(50.4)/ 59(56.2) | 100(59.5)/ 82(55.8) |  |  |
|  | G | 60(26.5)/ 56(26.7) | 77(22.9)/74(25.2) | 0.476 1.08[0.87-1.33] | 0.586 1.06[0.86-1.31] |
|  | T | 166(73.5)/154(73.3) | 259(77.1)/220(74.8) |  |  |
| rs10822974 | AA | 17(15.0)/ 19(18.1) | 44(26.2)/35(23.8) | 0.046 0.68[0.46-0.99] | 0.532 0.89[0.62-1.28] |
|  | GA | 64(56.6)/ 52(49.5) | 84(50.0)/ 64(43.5) |  |  |
|  | GG | 32(28.3)/ 34(32.4) | 40(23.8)/ 48(32.7) |  |  |
|  | A | 98(43.4)/ 90(42.9) | 172(51.2)/ 134(45.6) | 0.051 0.83[0.69-1.00] | 0.519 0.94[0.78-1.13] |
|  | G | 128(56.6)/120(57.1) | 164(48.8)/ 160(54.4) |  |  |
| rs7903491 | GG | 19(16.8)/ 25(23.8) | 21(12.5)/ 25(17.0) | 0.096 1.39[0.94-2.05] | 0.276 1.23[0.85-1.77] |
|  | AG | 60(53.1)/ 45(42.9) | 80(47.6)/ 68(46.3) |  |  |
|  | AA | 34(30.1)/ 35(33.3) | 67(39.9)/ 54(36.7) |  |  |
|  | G | 98(43.4)/ 95(45.2) | 122(36.3)/118(40.1) | 0.104 1.17[0.96-1.41] | 0.264 1.12[0.92-1.34] |
|  | A | 128(56.6)/ 115(54.8) | 214(63.7)/176(59.9) |  |  |

**Supplemental Table S2. Genotypes and alleles distributions of five selected variants in FET and controls.** All FET patients were compared with total healthy controls. *P* values were determined using binary logistic regression adjusted for age and gender, and p values ＜0.05 indicate significant differences between two groups. Abbreviations: FET: family essential tremor; HC: healthy controls.

| Variants | Genotypes/ Alleles | FET（157） (%) | HCs (n=315) (%) | *p*  OR(95%CI) |
| --- | --- | --- | --- | --- |
| rs10937625 | CC | 2(1.3) | 15(4.8) | 0.106 0.72[0.48-1.07] |
|  | TC | 38(24.2) | 91(28.9) |  |
|  | TT | 117(74.5) | 209(66.3) |  |
|  | C | 42(13.4) | 121(19.2) | 0.100 0.84[0.69-1.03] |
|  | T | 272(86.6) | 509(80.8) |  |
| rs17590046 | CC | 0(0) | 4(1.3) | 0.617 0.87[0.51-1.50] |
|  | TC | 21(13.6) | 43(13.7) |  |
|  | TT | 136(86.6) | 268(85.1) |  |
|  | C | 21(6.7) | 51(8.1) | 0.611 0.93[0.71-1.22] |
|  | T | 293(93.3) | 579(91.9) |  |
| rs12764057 | GG | 9(5.7) | 18(5.7) | 0.987 1.00[0.71-1.40] |
|  | TG | 58(36.9) | 115(36.5) |  |
|  | TT | 90(57.3) | 182(57.8) |  |
|  | G | 76(24.2) | 151(24.0) | 0.987 1.00[0.84-1.18] |
|  | T | 238(75.8) | 479(76.0) |  |
| rs10822974 | AA | 28(17.8) | 79(25.1) | 0.339 0.87[0.65-1.16] |
|  | GA | 87(55.4) | 148(47.0) |  |
|  | GG | 42(26.8) | 88(27.9) |  |
|  | A | 143(45.5) | 306(48.6) | 0.336 0.93[0.81-1.08] |
|  | G | 171(54.5) | 324(51.4) |  |
| rs7903491 | GG | 24(15.3) | 46(14.6) | 0.301 1.17[0.87-1.58] |
|  | AG | 80(51.0) | 148(47.0) |  |
|  | AA | 53(33.8) | 121(38.4) |  |
|  | G | 128(40.8) | 240(38.1) | 0.307 1.08[0.93-1.25] |
|  | A | 186(59.2) | 390(61.9) |  |

**Supplemental Table S3. The alleles frequencies of the five selected variants in different populations.** Abbreviations: 1000G, 1000Genomes; CHB, Han Chinese in Beijing; CHS, Southern Han Chinese; NA, not available.

| Databases  or Studies | Population | rs10937625 | | rs17590046 | | rs12764057 | | rs10822974 | | rs7903491 | |
| --- | --- | --- | --- | --- | --- | --- | --- | --- | --- | --- | --- |
|  |  | C | T | C | T | G | T | A | G | A | G |
| dbSNP | All | 0.1949 | 0.8051 | 0.1550 | 0.8450 | 0.3055 | 0.6945 | 0.4589 | 0.5411 | 0.3732 | 0.6268 |
| 1000G | All | 0.195 | 0.805 | 0.155 | 0.845 | 0.306 | 0.694 | 0.459 | 0.541 | 0.373 | 0.627 |
| 1000G | European | 0.255 | 0.745 | 0.184 | 0.816 | 0.426 | 0.574 | 0.498 | 0.502 | 0.401 | 0.599 |
| **1000G** | **East Asian** | **0.131** | **0.869** | **0.097** | **0.903** | **0.244** | **0.756** | **0.501** | **0.499** | **0.592** | **0.408** |
| **1000G** | **CHB** | **0.170** | **0.830** | **0.063** | **0.937** | **0.272** | **0.728** | **0.471** | **0.529** | **0.568** | **0.432** |
| **1000G** | **CHS** | **0.105** | **0.895** | **0.110** | **0.890** | **0.243** | **0.757** | **0.542** | **0.476** | **0.586** | **0.414** |
| Mullar’s study | European | 0.2615 | 0.7385 | 0.2054 | 0.7964 | 0.3721 | 0.6279 | 0.4588 | 0.5412 | 0.4609 | 0.5319 |
| **Xiao’s study** | **Asian** | **0.172** | **0.828** | **0.0989** | **0.9011** | NA | NA | NA | NA | NA | NA |
| **Our study** | **Chinese** | **0.192** | **0.808** | **0.081** | **0.919** | **0.240** | **0.760** | **0.486** | **0.514** | **0.619** | **0.381** |
